# Front-line hotel employees mental health and quality of life post COVID-19 pandemic: The role of coping strategies

**DOI:** 10.1016/j.heliyon.2023.e16915

**Published:** 2023-06-02

**Authors:** Ibrahim A. Elshaer

**Affiliations:** a Management Department, College of Business Administration, King Faisal University, Al-Ahsaa, 31982, Saudi Arabia; b Hotel Studies Department, Suez Canal University, Ismailia, 41522, Egypt

**Keywords:** Mental health, Stress, Depression, Anxiety, Problem solving, Social support, Avoidance, Quality of life, Hotel employees, SEM

## Abstract

The tourism business has been severely impacted by the COVID-19 pandemic, resulting in economic and job losses due to travel restrictions and lockdown measures. Among those most impacted are tourism employees, who have experienced job insecurity, financial difficulties, and increased work-related stress. The pandemic has also produced a significant negative effect on both mental health and quality of life (QOL) of these employees, leading to high levels of anxiety, stress, and depression. This study aims to assess the impacts of three coping strategies (problem-focused, social support, and avoidance) on the mental health and quality of life of front-line hotel employees. Data were collected from 700 participants and analyzed using SPSS version 25 and structural equation modeling (SEM) and AMOS program version 24. Our study found that social support and problem-solving coping strategies were effective in mitigating the negative impacts of stress, depression, and anxiety, while avoidance coping strategy did not have a significant impact. The mental health consequences of stress, depression, and anxiety were found to reduce the quality of life of hotel employees. The study highlights the significance of developing and implementing effective coping strategies to support the mental health and well-being of tourism employees. The findings suggest that organizations should provide resources and support to address the mental health needs of their employees.

## 1 Introduction

The COVID-19 pandemic created a massive effect on the global tourism sector, resulting in significant job losses and financial instability for those involved in the industry. The pandemic has also contributed to a rise in mental health consequences such as anxiety, stress, and depression, among hospitality workers [1]. Hospitality is a high-pressure field [[1], [2], [3], [4], [5]], with frequent shifts, workloads, unfavorable working hours, close contact with customers, and intense emotional labor [6,7]. Front line hotel employees, specifically, work long hours, and are required to maintain a high level of customer service, and often face difficult customer interactions [4]. These factors can contribute to the development of mental health issues in hotel workers. One common mental health issue among hotel employees that is prevalent in the hotel industry is depression. Hotel employees are at risk of depression due to factors such as isolation, job insecurity, and financial instability. The high-stress work environment can also contribute to feelings of anxiety and depression [6].

As the hospitality sector slowly recovers, it is essential to investigate coping strategies that can help reduce the unfavorable outcomes of the COVID-19 pandemic on the mental health of hospitality people. Coping can be described as an individual's ever-changing mental and behavioral exerted efforts in controlling internal and/or external stimuli that are apparent as surpassing his/her own resources [8,9]. In this sense, coping is a process-oriented, context-dependent activity that is not tied to any desired or undesired outcome when it comes to stress [10]. Coping has been explored alongside occupational stress since the earliest studies in this field (e.g., Ref. [11]). A coping strategy is identified as a system that people have devised to handle an emotional and/or social condition that would else be unbearable [12]. Coping tactics are closely investigated in connection with stress, depression, and anxiety [[13], [14], [15], [16], [17], [18], [19]]. Not all coping strategies have the same effectiveness in reducing stress, depression, and anxiety and may not have the same relationship with stress [20]. One explanation for this is that individuals may use an inner psychological mechanism called the cognitive appraisal process to cope with stress [[8], [9], [10], [11]]. Consequently, the effects of the same stressor may differ among individuals due to this internal mechanism. The objective of this research is to examine how three primary coping techniques, namely, problem-solving, social support, and avoidance, affect the mental health of hotel workers in terms of negative aspects such as depression, anxiety, and stress. Additionally, the study examines how depression, anxiety, and stress impact the quality of life (QOL) of hotel employees.

## 2 Literature review and research framework

The concept of coping strategies (i.e., social support, problem-solving, and avoidance) has been widely studied in the field of psychology, particularly in relation to mental health and quality of life (QOL). Coping strategies are considered to be a key factor in managing depression, anxiety, and stress, which in turn can impact an individual's QOL.

A considerable amount of literature is dedicated to defining and evaluating coping strategies [[12], [13], [14],^14^,[20], [21], [22], [23]]. Coping strategies have been disparate and encompass active coping, behavioural or psychological disengagement, seeking social support, seeking instrumental support, acceptance, emotional ventilation, seeking, denial, religious soothing [15,20,22]. Amirkhan [20] criticized both inductive and deductive taxonomies for operationalizing coping strategies and developed a more commonly appropriate tool to measure coping strategies by amalgamating various coping strategies. Amirkhan [20] suggested three main coping strategies (social support, problem-solving, and avoidance), which are supposed to have universal applicability and are also well-supported theoretically.

According to Amirkhan [20], problem solving coping involves taking active steps to address stressors and is characterized by a “fight” response rather than simply recognizing the stressor's existence. On the other hand, the avoidance coping strategy is seen as a way to avoid stressors, akin to a “flight” response. Social seeking support, as a separate strategy, implies that humans seek out contact with others during difficult times for reasons beyond practical assistance, advice, or distraction. In the hotel industry, problem-solving coping represents an employee's propensity to take action and find solutions to meet the demands of their job. This might involve seeking out additional training or learning opportunities. Avoidance coping refers to a personal strategy of selectively ignoring unpleasant aspects of a situation to focus only on the positive aspects, allowing the real source of stress to fade from awareness. Seeking social support refers to an employee confiding their personal difficulties with family and friends in their social community as a coping strategy during a stressful situation [16].

According to various studies [14,[24], [25], [26], [27]], problem-solving has been identified as an efficient coping strategy for reducing stress, depression, and anxiety. This strategy can also be useful for tourism employees who encounter challenges in their work, such as managing customer expectations, adjusting to changes in policies and procedures, and coping with unpredictable work schedules. Problem-solving empowers employees to take control of their work situation, diminish feelings of helplessness, and enhance their capacity to adapt to changing circumstances. In this way, taking direct action to seek solutions for problems can help hotel staff view challenges as opportunities and potentially reduce stress, depression, and anxiety [16,28].

According to Amirkhan [20] social support seeking may result in more severe illness, but the majority of research shows that it helps alleviate personal stress, depression, and anxiety [[29], [30], [31],[31], [31], [32], [33]]. In the tourism industry, social support seeking can assist employees in managing their job-related challenges, such as managing customer expectations, adapting to policy and procedure changes, and coping with unpredictable work schedules. Social support seeking provides emotional and practical assistance, which can alleviate feelings of isolation, enhance morale, and build resilience among employees. However, there is a limited volume of research on the connection between social support seeking coping strategy and stress, depression, and anxiety amongst hotel employees. There is little empirical evidence to support a positive association between social support seeking and mental health consequences among hotel employees, although some studies suggest that social support seeking can reduce the relationship between conflict in balancing work and family responsibilities, and intensify the inverse relationship between feeling exhausted from work and being able to effectively fulfill family obligations [27,33].

Avoidance coping is a strategy in which individuals attempt to avoid or withdraw from situations or stimuli that are perceived as threatening or stressful [14]. Despite providing some temporary relief, avoidance is generally not an efficient coping strategy and can worsen stress, depression, and anxiety among tourism employees after the COVID-19 pandemic [34]. Research has indicated that avoidance can actually lead to more psychological distress, and as such, it is believed to increase stress, depression, and anxiety levels contrasted to social support and active problem-solving strategies. Tsaur and Tang [35] discovered that deliberately diverting attention away from a stressful situation resulted in increased negative effects of job stress on hospitality employees' well-being. ‘Sunny’ and Cheng [16] observed that using avoidance coping strategy has a positive relationship with depersonalization, and a lack of individual achievement, which are all job burnout dimensions. These findings imply that the avoidance coping strategy may not be an adequate way to reduce job stress among hotels employees. Blalock and Joiner [36] observed that avoidance coping strategy (cognitive) worsened the influence of negative life actions on symptoms of stress. Holahan et al. [37] investigated the impact of avoidance as a coping strategy in creating both acute and chronic life stressors around 10-year time in 711 men and 500 women, using a longitudinal research design. The findings indicate, four years later, that individuals who engaged in avoidance coping strategies at the beginning of the study experienced more chronic and acute life stressors, as well as depressive symptoms ten years later. Although there are only a few studies in the hospitality industry that support the idea that avoidance can worsen stress, depression, and anxiety, more research is needed to confirm this. The theoretical background of this research paper is based upon the stress and coping theory, and job-demands-resources framework. The stress and coping theory proposes that people who engage in effective coping strategies are more likely to encounter positive mental health outcomes, such as reduced stress and increased resilience. This theory posits that stress is a result of a person's subjective interpretation of an event, and that coping strategies are the cognitive and behavioral efforts made to manage the demands of stressful situations [38]. Effective coping strategies can help individuals manage stress and maintain positive mental health outcomes. The job-demands-resources model suggests that work-associated demands can have adverse effects on employees' mental health and well-being. However, other resources, such as social support and problem solving, can help employees cope with job demands and maintain positive mental health outcomes [39].

**Fig. 1:**
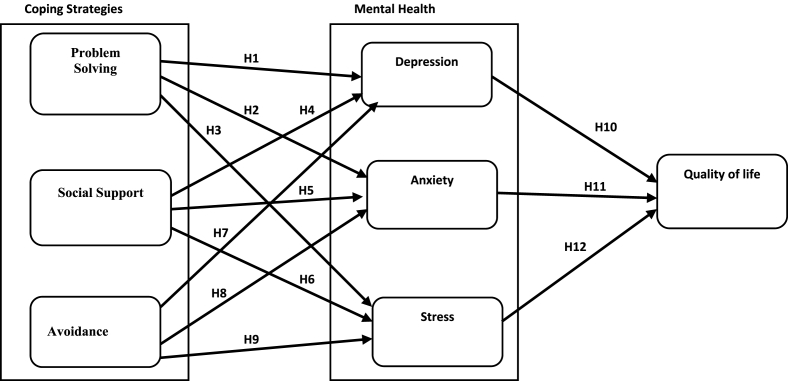
The study framework.

Although some studies have explored coping strategies in the tourism industry, few of them have focused on the specific coping strategies that hotel employees utilize to deal with the impact of the pandemic on their mental health and QOL. Understanding the effectiveness of coping strategies in the context of the COVID-19 pandemic can help inform the development of interventions to support the mental health and well-being of hotel employees in the aftermath of the pandemic. In light of the literature reviewed, and as seen in Fig. 1, we can suggest that.

(H1) Problem solving has a significant relationship with depression (as a dimension of employees' mental health).

(H2) Problem solving has a significant relationship with anxiety (as a dimension of employees' mental health).

(H3) Problem solving has a significant relationship with stress (as a dimension of employees' mental health).

(H4) Social support has a significant relationship with depression (as a dimension of employees' mental health).

(H5) Social support has a significant relationship with anxiety (as a dimension of employees' mental health).

(H6) Social support has a significant relationship with stress (as a dimension of employees' mental health).

(H7) Avoidance has a significant relationship with depression (as a dimension of employees' mental health).

(H8) Avoidance has a significant relationship with anxiety (as a dimension of employees' mental health).

(H9) Avoidance has a significant relationship with stress (as a dimension of employees' mental health).

The COVID-19 pandemic has caused anxiety, stress, and depression which have a significant influence on people's QOL, a measure of overall well-being and life satisfaction (World Health Organization [40]. Previous studies have demonstrated that stress, depression, and anxiety are correlated with lower QOL [27,41,42]. In the tourism industry, employees may experience high levels of stress due to job insecurity, reduced income, and uncertainty about the future of the industry, as well as social isolation and disrupted daily routines, leading to depression and anxiety. It is essential to study the impact of these mental health issues on the QOL of tourism employees since the industry is a crucial resource of income for several individuals and communities. A recent study in a popular tourist destination found that tourism employees experienced a decline in QOL after COVID-19, with high levels of stress, depression, and anxiety significantly predicting lower QOL [27,43]. The negative effects were more pronounced in female employees, those with lower education levels, and those in lower-income jobs. Therefore, targeted interventions are needed to support the mental health and well-being of tourism employees, especially those in vulnerable groups. We can, therefore, as seen in Fig. 1, propose that.

(H10) Depression (as a dimension of employees' mental health) has a significant association with employees' QOL.

(H11) Anxiety (as a dimension of employees' mental health) has a significant association with employees' QOL.

(H12) Stress (as a dimension of employees' mental health) has a significant association with employees' QOL.

## 3 Materials and methods

### 3.1 Sampling

The individuals who participated in this research were employees who worked full-time in the front office of luxury five-star hotels located in Egypt. After the COVID-19 pandemic, they were required to cope with their uncertain emotions, assess the disruptive challenges, identify the most effective strategic plan for the situation, and seize opportunities to help their organizations withstand and recover from the crisis. An expert firm was employed to reach out to a total of 200 five-star-hotel between December 2021 and January 2022, using the contact details from the Egyptian Hotel Guide [44] provided by the Egyptian Tourism Organisation. To prevent any upper or lower estimation of hotels numbers, each organization was given five questionnaires, resulting in 1000 questionnaires being distributed. Out of these, 700 completed questionnaires were obtained, which amounted to a response rate of 70%. The current study had a sufficient sample size of 700 participants for SEM testing, as it met Nunnally and Bernstein [45] criterion of at least ten respondents per each variable in the employed scale items. Furthermore, the study met Hair et al.'s [46] requirement for a minimum sample size of 100–150 to obtain acceptable maximum likelihood estimation solutions. Even though Krejcie and Morgan [47] recommend a sample size of 384 when the population surpasses 1,000,000, our study used a sample size of 700, exceeding this guideline. The significant benefit of such a large sample size is that it allows for advanced data analysis techniques like SEM to be used, enabling the successful investigation of the research variables' interdependence assumptions in our study.

Taking part in the survey was entirely optional and anonymous, and all measures were taken to safeguard the data confidentiality. To ensure the anonymity of the respondents, all identifying details were removed from the overtly available evaluation. Moreover, questions that asked for sensitive information such as age, name, and hotel name were optional.

### 3.2 Scale development

The study utilized a questionnaire with multiple questions to evaluate different concepts, as outlined in Table 1. The study employed the “Satisfaction with Life Scale” (SWLS) introduced by Diener et al. [48], which consists of five items, to measure the quality of life (QOL). The SWLS assesses an individual's general cognitive evaluation of their overall contentment with their life. Employees were requested to express the agreement level with statements reflecting life satisfaction, such as “My life is ideal in many ways” and “I am content with my life,” using a scale of 1 (indicating the lowest level of agreement) to 7 (indicating the highest level of agreement). The questionnaire showed an internal consistency of 0.963 in the present study.

**Table 1:** Results of CFA.

Latent	Observed	FL	α	CR	AVE	MSV
**Problem Solving**	Attempt to find a solution to the problem.	.840	0.948	0.947	0.781	0.157
	Make a deliberate plan of action instead of acting impulsively.	.810				
	Consider all feasible options before making a decision.	.940				
	Set specific objectives to manage the circumstance.	.959				
	I will attempt various approaches to address the issue until I come across one that proves successful.	.860				
**Social Support**	Share my concerns and anxieties with a trusted friend or family member.	.854	0.947	0.948	0.786	0.039
	Look for support from those who are familiar with me.	.859				
	Discuss the situation with others because it provides a sense of relief.	.933				
	Receive empathy and comprehension from friends who are experiencing similar issues.	.950				
	Consult a friend for suggestions on how to improve the situation.	.901				
**Avoidance**	Relate to fictional characters in books or movies.	.963	0.939	0.944	0.849	0.465
	Increase the amount of time spent watching television.	.899				
	Engage in more outdoor activities or games than usual.	.901				
**Depression**	I was unable to feel any positive emotions whatsoever.	.868	0.960	0.961	0.777	0.221
	It was challenging for me to motivate myself to do anything.	.863				
	There was a sense of hopelessness about the future.	.826				
	I felt sad and low-spirited.	.877				
	My self-worth was diminished.	.918				
	I couldn't muster any enthusiasm for anything.	.914				
	Life appeared to be without purpose or significance.	.902				
**Anxiety**	I noticed that my mouth was dry.	.791	0.970	0.970	0.821	0.465
	I had trouble breathing, such as rapid breathing or feeling breathless even when not physically exerting myself.	.795				
	I was anxious about situations where I might panic and embarrass myself.	.909				
	I experienced a sensation of being close to having a panic attack.	.967				
	I felt scared without any logical explanation.	.950				
	My hands were shaking.	.977				
	I was conscious of my heart's activity even though I wasn't physically exerting myself, such as feeling my heart rate increase or skip a beat.	.933				
**Stress**	I struggled to relax and unwind.	.943	0.972	0.972	0.835	0.151
	I tended to overreact to situations.	.941				
	I frequently became agitated.	.859				
	I was intolerant of any distractions or interruptions that hindered me from completing tasks.	.848				
	I felt overly sensitive and touchy.	.945				
	I expend a lot of nervous energy.	.971				
	I found it challenging to calm down	.880				
**Quality of life**	For the most part, my life is perfect.	.885	0.963	0.964	0.841	0.221
	I am content with my life.	.916				
	My circumstances are outstanding.	.924				
	Until now, I have achieved the significant things I desired in life.	.923				
	If I had the opportunity to live my life again, I would modify very little.	.937				
Model fit: (χ2 (681, N = 7000) = 2808.444, p < 0.001, normed χ2 = 4.124, RMSEA = 0.0251, SRMR = .024, CFI = 0.939, TLI = 0.925, NFI = 0.926, PCFI = 0.771 and PNFI = 0.758).						

The second scale employed in the study assessed the mental health of employees, specifically measuring depression (α = 0.960), anxiety (α = 0.970), and stress (α = 0.972). The scale consists of 21 items and is suitable for use in different context (industry and country). It is designed to measure an individual's negative emotions experienced during the most recent week, with each subscale comprising seven items. Employees were asked to evaluate how closely each item relevant to them during the past week using a Likert four-level scoring system, ranging from 0 to 3, where 0 indicates no conformity and 3 indicates a high level of conformity [49]. The higher the score, the more significant the level of negative emotions reported by the participant. In our study, the third scale used assessed employees' primary coping strategies, which include problem-solving (consisting of five items with a reliability of α = 0.948), social support (comprising five items with a reliability of α = 0.947), and avoidance (comprising three items with a reliability of α = 0.939), as suggested by Amirkhan [20].

Ten professionals in the Egyptian tourism industry and six academic professors in tourism colleges were asked to review the survey to ensure that the questions are simple, clear, necessary and appropriate. No significant changes were required during this process. To assess the questionnaire reliability, Cronbach's alpha (α) scores were employed. The values, as presented in Table 1, exceeded the threshold value of 0.7 suggested by Nunnally and Bernstein [45].

Since the obtained data were from a self-reporting survey method (Lindell & Whitney, 2001), several cautions were implemented to avoid the possible common method variance (CMV). During the questionnaire design process, several procedures were implemented to reduce the impact of CMV, as suggested by Ref. [50]. For instance, the dependent items were presented before the independent questions [51,52], and the confidentiality and anonymity of the respondents were ensured. To further investigate the potential issue of common method variance (CMV), Harman's-single-factor assessment was conducted using SPSS software v25, and Exploratory factor analysis (EFA) was conducted on all the indicators (dependent and independent variables). The sum of extracted factors was restricted to the value of 1 without using any rotation method. The results showed that only one dimension was extracted, which accounted for 35% of the variance, indicating that this factor did not explain a significant portion of the variance. Therefore, we can imply that CMV was not a problem in our study, as argued by Podsakoff et al. [50].

### 3.3 Data analysis methods

Various data analysis procedures were used in our research, including preliminary analysis to address normality, missing data, and sample size determination; descriptive examination to summarize employees’ characteristics, means, and standard deviations; and finally, multivariate analysis, including confirmatory factor analysis (CFA), and structural equation modeling (SEM). SEM was utilized as the primary analytical method due to its distinctive ability to integrate linear regression and factor analysis to simultaneously evaluate and test the intricate relationships among our model latent multidimensional factors [52]. Moreover, SEM can assess the direct and indirect causal relationships between the study variables while accounting for measurement errors associated with the imperfect measurement of variables [53,54].

In order to evaluate the goodness of model fit in SEM, the study used specific criteria recommended by Refs. [52,53,55], including: χ2/df (<0.5), SRMR (<0.05), RMSEA (<0.05), CFI (>0.90), NFI (>0.90), IFI (>0.90), TLI (>0.90), PCFI (>0.5), and PNFI (<0.5). The analysis was conducted using SPSS version 25 and AMOS version 24 software.

## 4 Study results

### 4.1 Descriptive and preliminary analysis

The data analysis process involved using SPSS version 25 to determine the lowest and highest values for missing data, which was found to be minimal (less than 5%) and not a significant concern. Skewness and kurtosis values were also evaluated to assess the normal distribution of scores, which showed no results outside the normal range of −2 to +2. The study consisted of 700 participants who were all employed full-time in five-star-hotels, with 70% being male and 30% being female. The vast majority (65%) were espoused, and most (60%) had completed a college degree. Approximately 71% of respondents had been with their organization for over five years. The mean scores were between 2.11 and 5.96, while standard deviation scores were adequate and were between 0.988 and 2.96, indicating that the data was more widely dispersed and less concentrated around its mean.

With regard to multicollinearity, the correlation coefficient in SPSS was utilized to validate the relationships between the variables. The Pearson r values between the variables were found to be within the range of −0.21 to 0.83, with no correlation exceeding 0.9. This suggests that there is no multicollinearity among the variables, as per Tabachnick and Fidell's [56] guidelines.

### 4.2 Multivariate analysis

#### 4.2.1 Confirmatory factor analysis (CFA)

To estimate our scale validity (convergent and discriminant), a first-order CFA was performed using Maximum Likelihood (ML) Estimation. The results of the first-order CFA indicated that the model fit well, as shown in Table 1. To measure the construct reliability, Cronbach's alpha values and composite reliability (CR) were used. The CR values for the seven measures employed were: QOL (0.963), depression (0.961), anxiety (0.970), stress (0.972), problem-solving (0.947), social support (0.948), and avoidance (0.944). All values surpassed the referenced cut-off level of 0.70, indicating adequate internal-consistency as argued by Nunnally and Bernstein [47]. Moreover, the data suggested that the scales had convergent validity for two main reasons. Firstly, the factor loadings (FL) of all reflective items were significant with values higher than 0.5 as suggested by Podsakoff et al. [50], and ranged from 0.802 to 0.977 (Table 1), Secondly, the “average variance extracted” (AVE) scores for all employed factors, namely QOL, depression, anxiety, stress, problem-solving, social support, and avoidance, were 0.841, 0.777, 0.821, 0.835, 0.781, 0.786, and 0.844, respectively (Table 1). All of these values surpassed 0.50, demonstrating adequate convergent validity [55]. In addition, the “maximum shared variance” (MSV) values were found to be lower than the AVE values (Table 1), indicating that the constructs had good discriminant validity, according to Hair et al. [55].

Table 2 displays the outcomes of the Fornell-Larcker matrix assessments, which indicate that the squared AVE values for each latent variable exceed the inter-variable correlation coefficients. This finding confirms that the constructs are distinct from one another, providing additional proof of adequate discriminant validity [57].

**Table 2:** Fornell-Larcker matrix results.

	I.	II.	III.	IV.	V.	VI.	VII.
I. Stress	**.914**						
II. Problem Solving	.210	**.884**					
III. Social Support	.104	.051	**.886**				
IV. Avoidance	.388	.396	.006	**.921**			
V. Depression	−.112	.031	.107	−.092	**.882**		
VI. Anxiety	.347	.176	.124	.682	−.089	**.906**	
VII. Quality of life	−.216	−.103	.198	−.297	.470	−.233	**.917**

#### 4.2.2 Structural equation modeling results

**Fig. 2:**
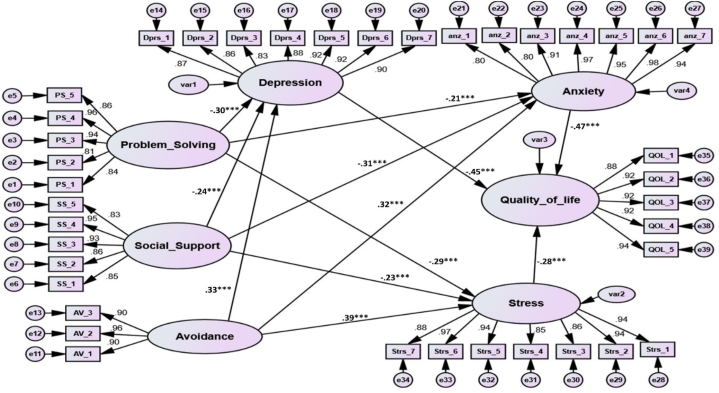
The Study structural model as an output from AMOS.

Our study used a confirmatory methodology, beginning with a review of existing literature to create a theoretical model. Empirical data was then collected to evaluate if it supported the previously established model [54]. The fit of the structural proposed model was evaluated by comparing it to a set previously determined standard as suggested by Refs. [52,53,55]. The findings from the SEM analysis revealed that the data aligned well with the theoretical model, as demonstrated in Table 3. After confirming that the model fit met the necessary criteria, the research hypotheses were evaluated. In the structural suggested model, each link connecting the latent variables represented a distinct hypothesis (as depicted in Fig. 2). The study posited a total of twelve hypotheses, all of which were subjected to scrutiny through an analysis of the path coefficients and associated p-values. The results demonstrated that all twelve hypotheses were supported, and further details regarding these findings are elaborated upon in the following paragraphs. (Table 3).

**Table 3:** Hypotheses results.

‵		Research model			
		β	T-value	SMC	Results
H1	Problem Solving→Depression	−.30***	- 7.256	–	Supported
H2	Problem Solving→Anxiety	−.21***	−4.511	–	Supported
H3	Problem Solving→Stress	−.29***	−4.284	–	Supported
H4	Social Support→Depression	−.24***	−3.898	–	Supported
H5	Social Support→Anxiety	−.31***	−6.154	–	Supported
H6	Social Support→Stress	−.23***	−4.599	–	Supported
H7	Avoidance→Depression	.33***	5.148	–	Supported
H8	Avoidance→Anxiety	.32***	4.174	–	Supported
H9	Avoidance→Stress	.39***	10.056	–	Supported
H10	Depression→Quality of life	−.45***	−10.159	–	Supported
H11	Anxiety→Quality of life	−.47***	−12.184	–	Supported
H12	Stress→Quality of life	−.28***	−4.001	–	Supported
Quality of life				.50	–
Model fit: (χ2 (690, N = 7000) = 3135.36, p < 0.001, normed χ2 = 4.544, RMSEA = 0.0261, SRMR = .0251, CFI = 0.935, TLI = 0.923, NFI = 0.921, PCFI = 0.777 and PNFI = 0.764).

The results indicated that utilizing problem-solving as a coping strategy was significantly linked to lower levels of depression (β = -0.30, t = − 7.256, p < 0.001), anxiety (β = -0.21, t = − 4.511, p < 0.001), and stress (β = -0.29, t = − 4.284, p < 0.001), confirming the validity of hypotheses 1, 2, and 3. Similarly, the utilization of social support as a coping mechanism was associated with significantly lower levels of depression (β = -0.24, t = − 3.898, p < 0.001), anxiety (β = -0.31, t = − 6.154, p < 0.001), and stress ((β = -0.23, t = − 4.599, p < 0.001), providing support for hypotheses 4, 5, and 6. Conversely, utilizing avoidance as a coping mechanism was found to have a positive and significant association with higher levels of depression (β = 5.148.256, p < 0.001), anxiety (β = 0.32, t = 4.174, p < 0.001), and stress ((β = 0.39, t = 10.056, p < 0.001). Therefore, hypotheses 7, 8, and 9 were supported. Additionally, the employee's quality of life was found to be adversely and significantly affected by depression (β = -0.45, t = − 10.159, p < 0.001), anxiety (β = -0.47, t = − 12.184, p < 0.001), and stress (β = -0.28, t = − 4.001, p < 0.001), which supports hypotheses 10, 11, and 12.

## 5 Discussion and implications

The COVID-19 pandemic has had a significant influence on the tourism business, with hotel employees facing a range of challenges. These challenges include job insecurity, reduced working hours, increased workload, and health concerns, all of which have the potential to negatively impact employees' mental health. Coping strategies are an essential aspect of maintaining good mental health including decreasing stress, anxiety, and depression. These negative impacts on mental health may also affect employees' quality of life. The current study investigated the impact of three main coping strategies (problem solving, social support, and avoidance) on hotel employees mental health negative aspects (depression, anxiety, and stress). The study further investigated the impact of depression, anxiety, and stress on hotel employees QOL.

The findings of our study found that problem solving coping strategy was linked with lower levels of depression, anxiety and stress among hotel employees. These results are consistent with [25,27]. These results infer that problem-solving coping strategies are an essential component of an individual's ability to deal with stressors effectively. It involves identifying the problem, generating and evaluating solutions, and implementing a solution to address the issue at hand. This approach is linked to better mental health outcomes among hotel employees. Furthermore, the current study provides evidence that social support coping strategy was associated with lower levels of depression, anxiety and stress among hotel employees. These results are in accordance with Cohen and McKay [30]; McClure and Moore [31]; Mossakowski and Zhang [32]; and Karatepe [33]. The findings suggest that hotel employees who have access to social support may be better equipped to manage stressors and experience fewer negative mental health outcomes.

On the other hand, the study results found that avoidance coping strategy was associated with a higher level of depression, anxiety and stress among hotel employees which is consistent with the results of Tsaur and Tang [35], Holahan et al. [37], Sunny’ Hu and Cheng [16]. Using this maladaptive coping strategy may provide temporary relief from stressors, but it does not address the root cause of the problem and could lead to negative mental health outcomes in the long run.

Additionally, our findings found that depression, anxiety, and stress are common mental health problems among tourism employees that can negatively impact their QOL post COVID-19 pandemic. These results are in accordance with the previous study results such as those by Dhaheri et al. [43]; Karatepe, Saydam, and Okumus [27]. The pandemic has led to a significant decrease in travel, causing many hotel employees to lose their jobs or experience reduced work hours and financial insecurity. This has led to a rise in mental health problems among hotel employees, which can negatively impact their quality of life. Employers in the tourism industry should prioritize the mental health and well-being of their employees by providing resources and support for mental health concerns, addressing job insecurity and financial difficulties, and promoting a positive work environment that supports employee well-being.

The current paper provides various theoretical and practical implications. Regarding the theoretical one, this research paper is focused on the ways in which coping strategies impact hotel employees' mental health and QOL post-COVID-19 pandemic. This research paper provides insights into the coping strategies employed by hotel people to manage stressors associated with their job. This research paper emphasizes the importance of adaptive-coping strategies, such as seeking social support, and problem-solving in promoting positive mental health outcomes in hotel employees. Maladaptive coping strategies, (avoidance), are correlated with negative mental health outcomes and decreased QOL. The findings of this research highlight the importance of providing employees with coping skills training to promote the use of adaptive coping strategies and reduce the use of maladaptive coping strategies. It's worth noting that, in Arabic culture, avoidance coping may be used to maintain social harmony, avoid confrontation, or preserve family honor. However, while avoidance coping may be culturally acceptable in some situations, it can have negative impacts on mental health and QOL.

The practical contributions of this research paper are focused on the ways in which hotel management can promote effective coping strategies and positively impact mental health outcomes for their employees post-COVID-19 pandemic. Designing an adequate employees training program can be a strategy that might be used to promote certain coping strategies to manage depression, stress, and anxiety and improve the QOL. This training could include strategies for stress management, time management, and self-care. For example, hotel employees could be trained in relaxation techniques, such as deep breathing or mindfulness meditation, to manage stress. Time management strategies could include tips for prioritizing tasks and managing time effectively. Self-care strategies could include recommendations for physical exercise, healthy eating, and sleep hygiene. Providing employees with coping skills training can help them better manage the stressors associated with their job and promote positive mental health outcomes. Workplace support is also identified as a critical factor in promoting positive mental health outcomes in hotel employees. This support could include flexible work arrangements, paid time off, and mental health resources. For example, hotel management could offer flexible work schedules or remote work options to help employees manage work-life balance. Paid time off could be offered for employees to take a break and recharge when needed. Mental health resources, such as employee assistance programs or access to mental health-professionals, could be provided to help employees manage stress and maintain positive mental health outcomes. Providing employees with workplace support can help them better manage the demands of their job and promote positive mental health outcomes. Finally, soliciting employee feedback is identified as a critical component of identifying areas where additional support or resources are needed. Hotel management can solicit feedback from employees through surveys or focus groups to understand their experiences and identify areas for improvement. This feedback can be employed to advise the development of coping skills training programs, workplace support initiatives, and mental health resources. Soliciting employee feedback can help hotel management better understand the needs of their employees and promote positive mental health outcomes.

## 6 Conclusion and limitations

The COVID-19 pandemic has had a significant impact on the tourism business, particularly on hotel employees who have been on the front lines of the pandemic. Coping strategies have been identified as a critical factor in maintaining mental health and QOL during and after the pandemic. CFA was conducted to test the scale validity and reliability. SEM was employed as the main data analysis technique. The paper found that two main coping strategies (social seeking support and problem solving) can decrease the negative impact of depression, anxiety, and stress of hotel employees post COVID-19 pandemic. While avoidance coping strategy was correlated with a higher level of stress, depression and anxiety among hotel employees. In return the quality of life among hotel employees was negatively impacted by lower-level mental health symptoms (depression, anxiety, and stress).

When interpreting the current results, it is critical to take into account various limitations of this research paper. The study has certain limitations regarding the establishment of causal connections between coping strategies, mental health outcomes, and QOL, as it is a cross-sectional study. To gain a better understanding of the relationships between these variables, it is necessary to conduct longitudinal studies over time. Additionally, the study relies on self-reported data, which may be influenced by social desirability bias and other response biases. Objective measures could be used in future studies to reduce bias. Furthermore, the study was conducted in a specific context (i.e., hotel industry) and with a specific sample (i.e., hotel employees), which reduces the generalizability of the findings to other industries or populations. Longitudinal studies could also be conducted to establish the temporal relationships between coping strategies, mental health outcomes, and QOL.

## Funding

The authors acknowledge the Deanship of Scientific Research at King Faisal University for the financial support under GRANT 3500).

## Institutional review board statement

The study was conducted according to the guidelines of the Declaration of Helsinki and approved by the deanship of scientific research ethical committee, King Faisal University (project number: GRANT 3500, date of approval: 15 January 2023).

## Informed consent statement

Informed consent was obtained from all subjects involved in the study.

## Author contribution statement

Ibrahim Elshaer: Conceived and designed the experiments; Performed the experiments; Analyzed and interpreted the data; Contributed reagents, materials, analysis tools or data; Wrote the paper.

## Data availability statement

Data will be made available on request.

## Additional information

Supplementary content related to this article has been publish online at [URL].

## Declaration of competing interest

The authors declare that they have no known competing financial interests or personal relationships that could have appeared to influence the work reported in this paper.
