# Supplementary material for: Front-line hotel employees mental health and quality of life post COVID-19 pandemic: The role of coping strategies
Source: Heliyon. 2023 Jun 2;9(6):e16915. doi: 10.1016/j.heliyon.2023.e16915 (PMC10234689; doi:10.1016/j.heliyon.2023.e16915)
Supplement: Multimedia component 1 [file mmc1.pdf]

Questionnaire briefing

Thank you for agreeing to undertake this survey. I am Ibrahim Elshaer, Professor of strategic management, KFU, school of Business. This survey is about investigating the coping strategies that can be employed to improve mental health and quality of life post COVID-19 pandemic. Your response will not be identified by name, or any other means, and will be completely anonymous and confidential.

I am interested in your own personal opinion and there are no "right" or "wrong" answers. However, it is important for you to be as precise and honest in your answers as possible. Please respond to all the questions even if you notice that some questions are very similar .

This survey will take approximate 10-15 minutes to complete. You have the right to withdraw from completing this survey at any point of time. If you withdraw, your response will be deleted and not be used in this research. Only researchers associated with this project will have access.

Thank you once again for taking the time to complete the questionnaire.

,Yours Sincerely

Ibrahim Elshaer

After reading the above briefing, by clicking on “I agree” button below, I am agreeing to give my consent to participate in this research study.

I agree ☐ Disagree ☐

The following information is needed for classification purposes only. For each item, please choose the appropriate answer.

- **Gender**
  Female ☐ Male ☐
- **Marital status**
  single ☐ married ☐ widowed ☐ divorced ☐ separated ☐
- **Working Experience**
  Less than a year ☐ from two to five years ☐ more than five years ☐
- **What is your highest level of completed education?**
  Less than a high school graduate ☐ Undergraduate degree (bachelor's degree) ☐
  High school graduate ☐ Postgraduate degree (master's or Doctoral degree) ☐

**Kindly evaluate how closely each item relevant to you during the past week where 0 indicates no conformity and 3 indicates a high level of conformity**				
	0	1	2	3
**Depression**				
I was unable to feel any positive emotions whatsoever.				
It was challenging for me to motivate myself to do anything.				
There was a sense of hopelessness about the future.				
I felt sad and low-spirited.				
My self-worth was diminished.				
I couldn't muster any enthusiasm for anything.				
Life appeared to be without purpose or significance.				
**Anxiety**				
I noticed that my mouth was dry.				
I had trouble breathing, such as rapid breathing or feeling breathless even when not physically exerting myself.				
I was anxious about situations where I might panic and embarrass myself.				
I experienced a sensation of being close to having a panic attack.				
I felt scared without any logical explanation.				
My hands were shaking.				
I was conscious of my heart's activity even though I wasn't physically exerting myself, such as feeling my heart rate increase or skip a beat.				
**Stress**				
I struggled to relax and unwind.				
I tended to overreact to situations.				
I frequently became agitated.				

I was intolerant of any distractions or interruptions that hindered me from completing tasks.				
I felt overly sensitive and touchy.				
I expend a lot of nervous energy.				
I found it challenging to calm down				

Please identify the extent to which each of the following items had been used to deal with a particular event where 1 refers to not at all, 2 means little and 3 means a lot.			
**Problem Solving**	1	**2**	**3**
Attempt to find a solution to the problem.			
Make a deliberate plan of action instead of acting impulsively.			
Consider all feasible options before making a decision.			
Set specific objectives to manage the circumstance.			
I will attempt various approaches to address the issue until I come across one that proves successful.			
**Social Support**			
Share my concerns and anxieties with a trusted friend or family member.			
Look for support from those who are familiar with me.			
Discuss the situation with others because it provides a sense of relief.			
Receive empathy and comprehension from friends who are experiencing similar issues.			
Consult a friend for suggestions on how to improve the situation.			
**Avoidance**			
Relate to fictional characters in books or movies.			
Increase the amount of time spent watching television.			
Engage in more outdoor activities or games than usual.			

Please express the agreement level with the following statements where 1 indicating the lowest level of agreement and 7 indicating the highest level of agreement.							
**Quality of life**							
	**1**	**2**	**3**	**4**	**5**	**6**	**7**
For the most part, my life is perfect.							
I am content with my life.							
My circumstances are outstanding.							
Until now, I have achieved the significant things I desired in life.							
If I had the opportunity to live my life again, I would modify very little.							

THANK YOU
